# Analysis of the Occurrence of Thermal Bridges in Several Variants of Connections of the Wall and the Ground Floor in Construction Technology with the Use of a Hemp–Lime Composite

**DOI:** 10.3390/ma12152392

**Published:** 2019-07-26

**Authors:** Przemysław Brzyski, Magdalena Grudzińska, Dariusz Majerek

**Affiliations:** 1 Faculty of Civil Engineering and Architecture, Lublin University of Technology, 40 Nadbystrzycka Str., 20-618 Lublin, Poland; 2 Fundamentals of Technology Faculty, Lublin University of Technology, 38 Nadbystrzycka Str., 20-618 Lublin, Poland

**Keywords:** hemp–lime, building partition, thermal bridge, thermal transmittance, thermal conductivity, condensation

## Abstract

This article analyses the connection of the two types of floors on the ground (floors on joists and self-supporting floors), with the external wall made of a hemp–lime composite for the occurrence of thermal bridges. Several factors that may affect the heat transfer in the junction were taken into account: the level of the floor on the ground, the wall thickness, the thermal conductivity of the composite, and the location of the timber frame construction. The technology of using hemp and lime is relatively new, and there is a lack of such analyses in the literature. The two-dimensional (2D) heat-transfer in the described construction joints was analyzed based on the finite-element method with the use of the THERM 7.4 software. The results were presented as averaged and linear thermal transmittance coefficients dependent on the above mentioned factors. The possibility of surface condensation was also checked. The differences in the values of the thermal transmittance of the junction between the two variants of ground floors reached around 0.13%–1.67% and the values of linear thermal transmittance factor reached approximately 2.43%–10.13%. The junctions with the highest floor level showed a decrease in the thermal transmittance value by about 3.00%–5.77% and in the linear thermal transmittance, by about 21.98%–53.83%, compared to the junctions with the lowest floor level. Calculations showed that almost all analyzed junctions are free from surface condensation causing mould growth, because the minimum temperature factors f_0.25_ were higher than 0.78 (except for junctions with the lowered floor levels). The junction with a floor on the timber joists showed better thermal parameters than the junction with a self-supporting floor in each of the analyzed variants. By increasing the level of floor insulation, it is possible to limit the thermal bridges and improve the thermal properties of the junction.

## 1. Introduction

**Figure 1:**
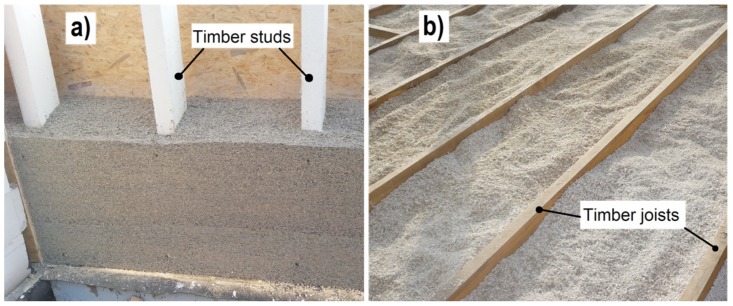
A timber frame wall (**a**) and floor of the joist construction (**b**) filled with a hemp–lime mixture.

The building and construction sector accounts for about 40% of world energy consumption and about 25% of global greenhouse gases emissions [1]. In order to reduce its impact on the natural environment, the European Union introduced a directive on energy performance of buildings in 2002 [2] (with amendment in 2010 [3] and 2018 [4]). Under this amendment, by the end of 2020, all buildings shall be characterized by nearly zero-energy consumption (“nearly zero-energy buildings”). The level of energy efficiency of a building depends to a large extent on the thermal insulation of the partitions used, limiting the fuel consumption for heating and decreasing the emissions of carbon dioxide into the atmosphere [5]. However, the energy consumption and greenhouse gas emission in the construction sector are also related to the phase of obtaining and production of building materials. Traditional insulation materials, such as extruded, expanded polystyrene, expanded polyurethane, or glass wool, are produced using non-renewable natural resources. These materials have high embodied energy in the range of 118.67–229.02 MJ eq per f.u. and high global warming potential in the range of 5.05–13.22 kg CO_2_ eq per f.u. [6]. It is, therefore, important to use thermal insulation materials that are environmentally friendly throughout their whole life cycle. The insulation materials with a low carbon footprint are often based on ingredients of plant origin. For example, industrial hemp shives, which are pieces of wooden parts of the hemp stem, are used for this purpose. They form a filler in a lime-based composite. The hemp–lime composite is used as filling for a wall with a load-bearing timber frame (Figure 1). In addition, the hemp–lime composite can be applied as a thermal insulation for floors (Figure 1) and roofs.

There are several steps for filling a partition with the composite: laying of the hemp–lime mixture in the formwork by hand, mechanical spraying of the permanent shuttering, laying of hemp–lime blocks, and assembly of large-size elements [7,8]. The composite is characterized by a low apparent density in the range of 256–627 kg/m^3^ [9,10], satisfactory insulating properties (an exemplary range of the thermal conductivity coefficient is 0.082–0.151 W/(m·K) [11]), high vapor permeability (a diffusion resistance coefficient of 5–6 [10], high mass absorptivity (110.8–134.5% for the composites with apparent density of 411.6–438.7 kg/m^3^) [12]), high total porosity in the range of 72%–80% for composites with an apparent density of 256–460 kg/m^3^ [9], low mechanical properties (compressive strength in the range of 0.23–0.85 MPa) [11,12], and a high thermal capacity (which for exemplary composites described in the literature was 1300 J/kg·K [10]). Due to the high hygroscopicity of the material, the walls made of the composite exhibit the ability to regulate the humidity level in the rooms [7,8]. In addition to the insulating function, the composite also has the function of stiffening the timber wall construction [13]. The lime binder, as a material with a high pH, protects the shives against biological corrosion and protects the composite against fire action [7]. In addition to hydrated lime, binder additives are used, accelerating the binding and improving the physical and mechanical properties of the composite. Examples of such additives are metakaolinite [12], zeolite [14], MgO cement [14], and ground granulated blast furnace slag [10]. In the construction industry, hemp fibers are also used as a thermal insulation material due to their low lambda value at a level of 0.04 W/(m·K) [15]. Hemp fibers and shives, as well as other cellulose materials [16,17], are also used as micro-reinforcements and fillers in mortars and plasters.

The thermal insulation materials (as well as plant based thermal insulations) produced nowadays are increasingly more effective. Nevertheless, there are places or surfaces in the building with increased heat transfer to the outside, such as thermal bridges, which can increase heat losses in the heating season by up to 30% [18,19]. These include, among others, structural elements cutting through the continuity of thermal insulation (balconies, columns), construction joints (such as a floor on the ground with an external wall or a roof with an external wall), and a change in the shape of building elements.

The share of bridges in heat losses mainly depends on the construction of the building and the insulating properties of the external partitions. Complicated shapes of the building envelope are often the cause of the occurrence of thermal bridges. Kosny et al. [20] proved that in some buildings, up to 50% of the elevation area consists of three-dimensional envelope structural details. A study by Ge et al. [21] showed that for a typical high-rise multi-unit residential building, a balcony cross-section area representing 4% of the total exterior wall may contribute up to 11% of the space heating energy consumption, depending on the thermal performance of the windows and the opacity of its walls. The research carried out by Citterio and co-authors [22] showed that the relative effect of thermal bridges on the annual heating energy demand varies by 7% in typical houses built in the seventies to 28% for modern high-quality houses. According to [23], structural junctions can increase heat transfer to 30%, which results in an increase in the energy demand for heating. Cappelletti et al. [24] showed, in turn, that the influence of thermal bridges on the energy needs of a building for space heating can be as high as 67% for a building with double-layer brick walls with *U* = 0.15 W/(m^2^K) in a typical Italian climate zone. The results of other studies [25] showed that the improvement of thermal bridges is an effective way to reduce the energy demand for heating (by 25% in the case of terraced houses and 17.5% in the case of semi-detached houses). Another study on the occurrence of thermal bridges in typical constructions showed that improved building envelope details minimizing thermal bridges can result in up to 10% energy savings, which is comparable to increasing the insulation levels and using triple-glazed windows [26].

Another unfavorable phenomenon related to thermal bridges is the change in the temperature distribution and its reduction on the surface of the partitions. The risk of surface condensation, interstitial condensation, and the growth of mould during the winter are increased. Thus, the air quality in the room is reduced [27]. The presence of moisture and mould is a threat to the health of residents [28,29]. Moulds produce allergens (substances that may cause allergic reactions), irritants, or even toxic substances. Inhaling or touching mould spores can cause an allergic reaction. They can also cause asthma attacks and other respiratory diseases. Methods for reducing the risk of mould growth in thermal bridges were investigated. Fantucci et al. [30] proved that the presence of a fine insulation rendering coat has a significant effect on mould growth risk reduction at the interior side of a vertical wall, i.e., a concrete slab junction.

The elimination of thermal bridges is very important in the case of walls made of materials based on plant components, because they are particularly exposed to biological corrosion [29,31]. The examples include such materials as straw, a mixture of clay and straw, and a hemp–lime composite. In natural building technologies, such as strawbale or hempcrete, a timber frame construction is used [8,32]. Timber elements are usually covered with a thermal insulation material. The thermal conductivity coefficient of pinewood is about 0.16 W/(m·K) [33] and does not significantly differ from the thermal conductivity coefficient of straw: 0.073 W/(m·K) [34] or a hemp–lime composite: 0.082–0.151 W/(m·K) [11]. The filling with the hemp–lime composite improves the stiffness of the timber frame construction, which means that a reduced cross-section of the columns can be applied, compared to the timber frame filled with mineral wool, where the number of horizontal or vertical stiffeners is limited [7]. However, at the points of construction junctions, there is a larger number of timber elements than in other parts of partitions. Therefore, these areas are potential thermal bridges.

In connection with the aforementioned problems, this article analyzes various junctions—a combination of an external wall, a foundation wall and the floor on the ground, made in timber technology with a fill in the form of a hemp–lime composite. The influence of several factors (the position of the wooden wall frame, wall thickness, the thermal conductivity of the composite, the floor level on the ground, and the type of floor construction) on the thermal properties (averaged thermal transmittance and linear thermal transmittance) of the junctions was checked. Using second order polynomial regressions with an interactions statistical model (built by the authors), the significance of the influence of a given factor on the results was assessed. The possibility of surface condensation was also estimated. The temperature distribution in the described construction joints was calculated using the THERM 7.4 software. Our own recipes for walls and floor hemp–lime mixes were used for the analyses. The literature describes thermal bridge analyses in the area of windows, ceilings, and wall studs in construction technology with the use of hemp–lime composite [35,36], but there is lack of analyses on the occurrence of thermal bridges in different variants of connections between the wall and the ground floor. Many junction configurations were considered to determine the effect of individual factors on thermal transmittance. The results presented in the article may be helpful in the design of buildings using this technology.

## 2. Materials and Methods

### 2.1. Materials Used in Calculations

In the conducted analyses, hemp–lime mixtures were applied in the walls, in the floor between the joists (loose mix), and in the self-supporting floor (as a dense layer of the floor). The recipes for the composites and their apparent densities (an average of three samples) are shown in Table 1.

A hemp–lime mixture with a minimum content of lime binder was used as the insulation of the floor based on timber joists. These ratios are proposed (with a different binder) [37] as roof insulation between rafters, but in practice, this binder is used in the same way between timber joists in the floor. In this case, the binder should only coat the shives to protect them against biological corrosion. It is not required to bind the shives with a binder, because the mixture performs only an insulating function. This mixture was referred to as “floor mix A”. Another mixture, referred to as “floor mix B”, containing a larger amount of binder (in order to increase strength), is used in self-supporting floors. The presented floor mix proportions (with a different binder) are used in practice [37]. A hemp–lime mixture was used as an insulating material in the wall; two variants, differing in the proportion of binder to shives, were employed. The binder: hemp shives ratios of the wall mixes result from our own experience and are also used in practice, depending on the expected thermal parameters. According to the previously conducted tests, the binder: filler ratio in “Wall mix A” turned out to be minimal, in which the mixture retained the appropriate viscosity, enabling the formation of composite samples. The choice of this proportion was also related to the expectedly low thermal conductivity of the composite. In turn, the binder: filler ratio in “Wall mix B” was selected to show the influence of lambda on the heat flow in the analysed junctions, guided by the literature [11,38] and our own research, which proved that the thermal conductivity coefficient of the composite increases along with the binder content.

The mixture consisted of hemp shives obtained from industrial hemp stalks Białobrzeskie (Podlaskie Konopie, Białystok, Poland), a lime binder made of hydrated lime (CL-90s class) (Lhoist, Tarnów, Poland), and water. In this case, the use of a binder-accelerating additive was not necessary due to the loose form of the non-loadbearing hemp–lime compound. The binder of other composites consisted of hydrated lime CL90s class (Lhoist, Tarnów, Poland), amounting to 75% by weight, gypsum (Atlas, Pińczów, Poland), making 15%, and pozzolan–metakaolin (Astra Polska, Gdańsk, Poland), making 10%. Gypsum was used in order to accelerate the setting process, and pozzolan was used to partially achieve the hydraulic properties of the composites.

### 2.2. Thermal Conductivity of Hemp–Lime Composites Used

**Figure 2:**
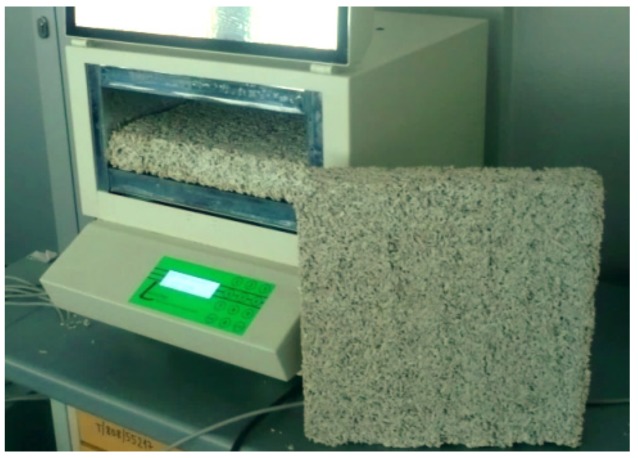
Heat flow meter—Fox 314 apparatus with the hemp–lime composite (Wall mix A) sample.

Thermal conductivity was measured in a FOX314 plate apparatus (TA Instruments, New Castle, DE, USA) consisting of a cooling and heating plate with a heat flux sensor, in accordance with the ISO 8302 method [39]. A thermal conductivity test of the composites was carried out on specimens with the following dimensions: 50 × 300 × 300 mm. The measuring setup with an exemplary sample is presented in Figure 2. The “Floor mix A” mix, due to its loose form, was placed in a frame made of expanded polystyrene. Before the tests, the samples were dried at a constant temperature of 50 °C.

The temperature set on the heating plate was 25 °C, while on the cooling plate it was 0 °C. The thermal conductivity test results were averaged over the results obtained from five specimens, the final coefficients are shown in Table 1. These parameters were used in the calculation of thermal bridges of the analysed junctions.

The obtained results for the wall and floor are comparable with others presented in the literature. In the technical sheet provided by the producer of the lime binders and shives [37], the thermal conductivity of the floor mixes used in self-supporting floors was 0.11 W/(m·K), while the thermal conductivity of the roof insulation mixes (which could also be used as the insulation of the floor based on timber joists) was 0.06W/(m·K). Benfratello et al. [38] presented the value of the thermal conductivity coefficient for a wall composite with apparent density of 377 kg/m^3^, equal to 0.089 W/(m·K). In other studies [40], a composite with a density of 330 kg/m^3^ was characterized by a lambda value of 0.078 W/(m·K). On the other hand, using a higher proportion of a binder to hemp shives (2:1 by weight), the composite with a density of 508 kg/m^3^ was characterized by a thermal conductivity of 0.117 W/(m·K) [10].

In the case of the wall mixes, the obtained thermal conductivity values were used in further computer analyses of thermal bridges as limit values for the thermal conductivity coefficient. In addition, indirect values from the 0.080–0.088 W/(m·K) interval, namely 0.082 W/(m·K), 0.084 W/(m·K), and 0.086 W/(m·K), which are likely to be achieved using a binder to filler ratio from 1.4:1 to 1.6:1, were used to illustrate the relationship between the thermal quality of a joint and the thermal conductivity of a wall material. The increase in the thermal conductivity value of the composite along with the binder content is confirmed in the literature [11,38].

### 2.3. Schemes Adopted for Calculation

**Figure 3:**
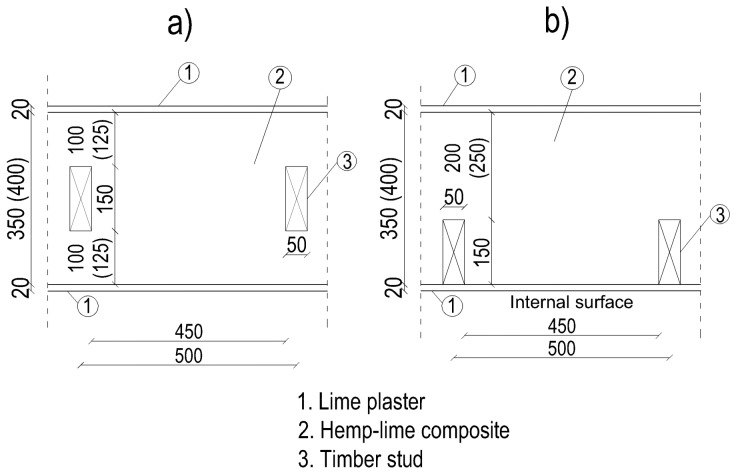
External walls with a timber frame construction located centrally with respect to the wall thickness (**a**) and at the inner side of the wall (**b**). Dimensions in mm.

The ground floor junction was modelled in several variants to analyse the occurrence of thermal bridges. The timber wall was placed on the foundation wall made of concrete blocks and a strip foundation. The timber load-bearing frame was located centrally in relation to the wall thickness, or it was placed on the inside of the wall. The frame consists of studs with a cross-section of 50 × 150 mm, spaced axially every 500 mm (Figure 3).

The thickness of the composite layer considered was 350 and 400 mm and the following markings were used:

- 350i—a wall with a 350 mm thick composite layer and a frame placed on the inside of the wall
- 350c—a wall with a 350 mm thick composite layer and with the frame placed centrally with respect to the wall thickness
- 400i—a wall with a 400 mm thick composite layer and a frame placed on the inside of the wall
- 400c—a wall with a 400 mm thick composite layer and with a frame placed centrally with respect to the wall thickness.

**Figure 4:**
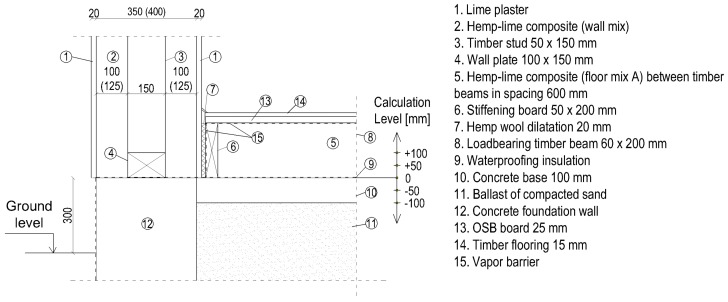
Ground floor junction with a floor on the ground with a structure in the form of timber joists. The scheme shows the assumed floor levels (−100, −50, 0, +50, +100). Dimensions in [mm].

The floor on the ground was designed in two ways. In the first one, the construction of the floor was made of timber joists, and the spaces between them were filled with a loose, lightweight mix of hemp–lime (floor mix A). This solution is illustrated in Figure 4.

**Figure 5:**
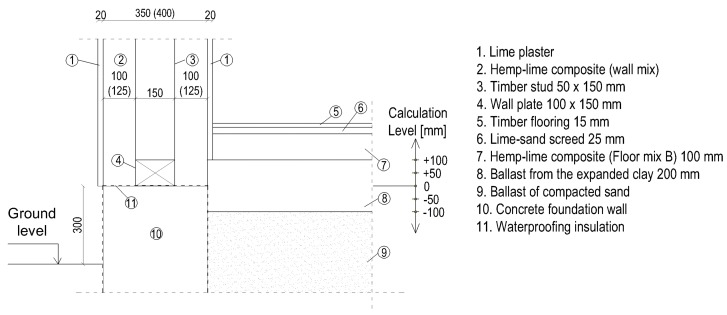
Ground floor junction with the floor on self-supporting ground. The diagram shows the assumed floor levels on the ground (−100, −50, 0, +50, +100). Dimensions in mm.

In the second method, loads are transferred to the subsoil using a layer of hemp–lime composite of a higher density (floor mix B), which also serves as thermal insulation. An additional thermal insulation layer (substructure) in the form of an expanded clay aggregate was designed to increase the effectiveness of thermal insulation. The layer of hemp–lime composite should not be too thick due to the limited possibility of drying. For this reason, a thickness of 100 mm was adopted, and the proper thermal insulation was ensured by an additional layer of expanded clay aggregate. When the layer of the hemp–lime mix is placed on the ballast of the drainage aggregate, the waterproofing insulation is not used [7,8]. The floor scheme is shown in Figure 5.

In the calculations, the level of the floor was changed. Five levels were considered: the base level 0 and the floor level raised by 50 mm and 100 mm, as well as the floor level reduced by 50 and 100 mm. These are marked as “−100”, “−50”, “0”, “+50”, “+100,” and shown in Figure 4 and Figure 5.

### 2.4. Simulation

#### The Basis of Calculations

The temperature distribution in the described construction joints was calculated with the use of the THERM 7.4 software (Lawrence Berkeley National Laboratory, Berkeley, CA, USA) [41], commonly used in the thermal evaluation of building partitions and construction joints [42,43,44].

The code was validated in [45] in accordance with the procedures of the standard [46], based on a comparison of the numerical results with a strict analytical solution. This is considered very accurate and is one of the most reliable methods of validation [47,48,49]. The validation of a 2D method consisted of modelling two cases (half of a rectangular column and a fragment of an insulated building element) and comparing the temperature distribution in the given points. All the results lie within the requested 0.1 K difference in temperature and 0.1 W/m difference in heat flow, and according to [45], the code can be classified as “a two dimensional high precision calculation method”.

Experimental validation of the models, to the knowledge of the authors, would not be possible. The connection of building partitions with the ground is too large to be examined in a climatic chamber or under any laboratory conditions. Measuring temperature and heat fluxes in a joint operating in real environment conditions would not give reliable results either, because of the outside temperature variations, making it impossible to maintain steady state conditions (even with a stabilized internal temperature), necessary for the evaluation of linear thermal transmittance coefficients.

In the code, two-dimensional heat transfer equations are solved numerically for specific elements using the finite element method. The modelling process consists of the following stages:

- Model definition (including geometry definition, assignment of material properties and boundary conditions);
- Mesh generation;
- Calculation of temperature and heat fluxes by the Finite Element Analysis Solver;
- Reporting of the post-processed results for the element.

The results are displayed in a graphic form as isotherm and heat flux outlines, allowing for the visual and qualitative evaluation of a thermal bridge. Information about the thermal transmittance coefficient (*U* [W/(m^2^·K)]), averaged for the whole element, can be used for further quantitative assessment of a joint, namely calculation of the linear thermal transmittance coefficient *ψ* [W/(m·K)].

The calculation procedure according to ISO 10211 [46] requires modelling of the element in such a way that it is extended at least 1 m or a triple thickness of the element away from the geometrical centre of the thermal bridge, in order to restore one-dimensional heat flow at the cut-off plane (treated as an adiabatic). In the described cases, the triple thickness of the element is the dominant criterion. The dimensions of the ground (together with the floor layers) should extend to 0.5·*B* inside the building and 2.5·*B* outside the building and below ground, where B is the width of the floor. In the pictures of the isotherms presented below, some of the partitions and the area outside the building and below ground were cut shorter, but this was only made for the clarity of the presentation.

**Figure 6:**
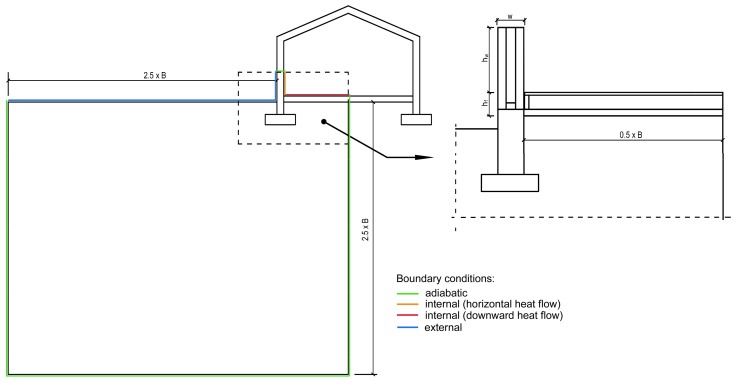
Dimensions of the partitions used in Equation (1): *w* = 0.39 or 0.44 m, *h_w_* = 3·w = 1.17 or 1.32 m, *h_f_* = 0.34 m, *B* = 8.00 m (recommended by [46]).

The linear thermal transmittance coefficient ψ was calculated according to EN ISO 10211 [46] with Formula (1) using external dimensions and, to compare the results, by means of Formula (2) using internal dimensions (Figure 6):

(1) $$\psi_{e}=L^{2D}-\left(h_{w}+h_{f}\right)\cdot U_{w}-\left(0.5\cdot B+w\right)\cdot U_{g}$$

(2) $$\psi_{i}=L^{2D}-h_{w}\cdot U_{w}-0.5\cdot B\cdot U_{g}$$

where *L*^2*D*^ is the thermal coupling coefficient obtained from the 2D analysis of the modelled element as a multiplication of the averaged thermal transmittance *U* and the joint’s length [W/(m·K)]; *h_w_* is the minimum distance from junction to the cut-off plane [m]; *h_f_* is the height of the top of the floor slab above the ground level [m]; *U_w_* is the thermal transmittance coefficient of the external wall (W/(m^2^·K)); *U_f_* is the thermal transmittance coefficient of the foundation wall (W/(m^2^·K)); *B* is the width of the floor [m]; *w* is the width of the external wall [m]; *U_g_* is the thermal transmittance coefficient of the floor [W/(m^2^·K)].

The values of the *U_w_* and *U_g_* coefficients were calculated in compliance with the ISO 6946 [50] and ISO 13370 standards [51] (accordingly). The layers that are not uniform (walls and floor parts consisting of timber studs and insulation between them) were initially modelled in THERM in order to obtain the equivalent thermal conductivity values used in the calculations and further modelling of the junctions. The dimensions of the floor were assumed to be 8 m × 12 m.

The thermal conductivity of the materials and elements used in modelling, together with the boundary conditions, are compiled in Table 2 and Table 3. The thermal conductivity value of materials other than hemp–lime were acquired from the ISO 10456 standard [33]. The absolute error of the numerical analyses (calculated as the absolute value of the difference between heat inflow and outflow divided by the mean heat flux through the joint) was in the range of 0.0% to 0.1%.

One of the limitations of the research is excluding, from the analyses, the influence of the foundation wall on the linear thermal transmittance. The wall construction was assumed to be made of concrete, which is a typical solution in buildings with a timber frame construction. It was not changed during the analyses, as the authors wanted to concentrate on other factors, such as construction of the partitions (walls and floor) and floor level.

### 2.5. Statistical Models

A lot of variables which may affect the results have been taken into account. The variables are:

- Type of the floor construction: the floor on timber joists or a self-supporting floor;
- Level of the floor—from −100 mm to +100 mm;
- Wall thickness—350 mm or 400 mm;
- Thermal conductivity of hemp–lime composite (wall mix)—from 0.080 W/(m·K) to 0.088 W/(m·K);
- Location of timber frame construction (centrally in relation to wall thickness, at the inner side of the wall).

Each of these factors has an effect on the thermal parameters of the junction, but it would be useful from a practical point of view to determine which factor exerts the greatest influence on thermal transmittance and linear thermal transmittance. Second order polynomial regressions with interactions were built (Equations (3) and (4)). The statistical model concerns the results of thermal transmittance value calculations and linear thermal transmittance values calculated on the basis of external dimensions:

*U* = *U*(*l*, *λ*, *w*, *f*) (3)

*ψ* = *ψ*(*l*, *λ*, *w*, *f*), (4)

where *U* is the thermal transmittance (W/(m^2^·K)); *ψ* is the linear thermal transmittance (W/(m·K)); *l* is the level of floor on the ground (−100 mm–100 mm); *λ* is the thermal conductivity of hemp–lime wall composite (0.08 W/(m·K)–0.088 W/(m·K)); *w* is the thickness of the hemp–lime layer in the wall (350 mm or 400 mm); *f* is the timber frame (two locations: the timber frame construction located centrally in relation to the wall thickness or at the inner side of the wall).

The final functional forms of models *U* and *ψ* are the effect of cutting off all the variables that were non-significant. In the models there are significant variables of higher orders. Therefore, it is not possible to unambiguously answer the question of which factor has the greatest impact on thermal transmittance [52]. Table 4 shows the models for the averaged thermal transmittance and linear thermal transmittance of both floor–wall junctions.

The parameters of both models for particular dependent variable are quite similar, so the impact of each independent variable is almost the same in both models. There is one exception in thermal transmittance models, where the effect of thermal insulation is twice as large in model (1) as in model (2). All four models are a very good fit. The percentage of the explained variance of dependent variables by predictors in all models is higher than 99%, which means that all functional forms of the considered relationship describes them very well. All insignificant effects were removed from models by a backward elimination technique. Global tests (F Statistic) are all significant, which means that there is a relationship between the response and the linear combination of the predictors. Very low values of a residual standard error show that thermal transmittance (linear thermal transmittance) is well described by the model [53].

Interpretation of all the above-mentioned models is difficult because of their nonlinear nature. However, the estimation of average mean effects of predictors hints at the nature of the relationship. The average mean effect of the timber frame is small but positive for all models. Thermal insulation has a positive effect in the model for thermal transmittance, but a negative one for linear thermal transmittance. The rest of predictors have a negative average mean effect on thermal transmittance and linear thermal transmittance.

All estimations of model parameters were performed with the ordinary least squares method in the R statistical environment [54]. The assumptions of the Gauss–Markov theorem for linear models were fulfilled.

### 2.6. Possibility of Water Vapour Condensation

External partitions should be designed taking into account the risk of surface condensation causing mould growth. Moisture condensation on the internal surfaces of the partitions may occur in places where the temperature is lower than the dew point temperature. Thermal bridges are susceptible to this phenomenon because of the local temperature drop connected with the increase of heat transfer. The condition of surface condensation occurrence was checked based on the ISO 13788 standard [55]. The design for the avoidance of mould growth requires that in the critical area, the temperature factor at the internal surface *f_Rsi_* is higher than the design temperature factor *f_Rsi,max_* appointed for the critical month (which is the month with the highest *f_Rsi,min_* value). Both of the factors should be calculated using the increased thermal resistance at the internal surface (*R_si_* = 0.25 (m^2^·K)/W):

(5) $$f_{0.25}=\frac{\theta_{si}-\theta_{e}}{\theta_{i}-\theta_{e}}\geq f_{0.25,max}=\frac{\theta_{si,min}-\theta_{e}}{\theta_{i}-\theta_{e}}$$

where *θ_si_* is the surface temperature in the critical area (°C), *θ_si,min_* is the minimum acceptable surface temperature (°C), *θ_i_* is the internal temperature (°C), and *θ_e_* is the external temperature (°C).

## 3. Results and Discussion

### 3.1. Heat Flow Analysis

**Figure 7:**
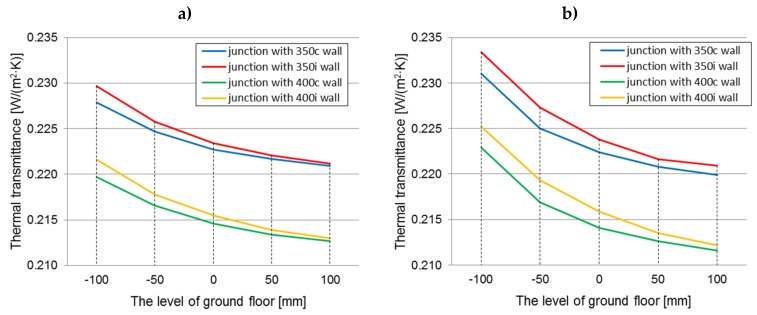
The averaged thermal transmittance of the ground junction in all construction variants of walls filled with hemp–lime composite (*λ* = 0.080 W/(m·K)) depending on the floor level on the ground: (**a**) floor on joists; (**b**) self-supporting floor.

The graphs (Figure 7a,b) show the changes in the average thermal transmittance coefficient for the ground junction with all the design variants of the analysed walls filled with hemp–lime composites characterized by a thermal conductivity coefficient of 0.08 W/(m·K), depending on the level of the floor ground. The average thermal transmittance is a value from the THERM models, describing the thermal transmittance averaged over the surface of the modelled elements (internal or external).

- The influence of the timber frame location on the thermal transmittance value within every floor level (constant thermal conductivity, 0.080 W/(m·K))

In both cases of the floor construction, better thermal parameters were exhibited by a junction with a timber frame placed centrally with respect to the wall thickness. In the case of the floor on joists, the thermal transmittance coefficients were lower by 0.0003 W/(m^2^·K)–0.0019 W/(m^2^·K), while for the self-supporting floor, the coefficients were lower by 0.0006 W/(m^2^·K)–0.0024 W/(m^2^·K), relative to junctions with the timber frame placed on the inside of the wall (taking into account both the wall thickness of 350 and 400 mm, but with the same value of *λ* = 0.080 W/(m·K)). Thermal insulation of the wall and floor maintains continuity over the entire length of the junction. The differences in the thermal transmittance factor are greater for the case of a self-supporting floor, but in both cases they decrease as the level of the floor increases. The differences caused by the two locations of the timber frame (considering the wall thickness of 350 and 400 mm) in the value of the thermal transmittance coefficient of the junction at the level “−100” are 0.79%–0.86% (floor A), 1.03%–1.04% (floor B), and 0.14% (floor A) and 0.28–0.45% (floor B) at the level “+100”.

The impact of the timber frame location is statistically significant. Examination of the edge effects proved that for a constant *λ* = 0.08 W/(m·K), and with varying other parameters, the influence of the timber frame location is significant and indicates lower thermal transmittance values for the junctions with a timber frame placed centrally with respect to the wall thickness.

- The influence of the floor level on the thermal transmittance value in every wall’s thickness and the location of the timber frame (constant thermal conductivity, 0.080 W/(m·K))

As the floor level is raised above the ground, the thermal transmittance value is reduced. This is related to an increase in the contact area between the floor and the wall thermal insulation. The differences between the thermal transmittance coefficient at “−100” and “+100” levels ranges from 0.0070 to 0.0086 W/(m^2^·K) (3.16–4.04%) for a junction with a floor based on joists and from 0.0111 to 0.0130 W/(m^2^·K) (5.05–6.13%) for a junction with a self-supporting floor, taking into account the wall thickness and the location of the timber frame. In both cases, the greatest difference is for the wall “350i” and the smallest is for the wall “400c”. The influence of the level of the floor on the thermal transmittance value of the junction is statistically significant for both types of floor construction.

- The influence of the type of floor construction (on joists or self-supporting) on the thermal transmittance value (constant: λ = 0.080 W/(m·K), a variable: wall thickness, location of the timber frame, and floor level)

A better thermal floor construction for the level below “0” is the floor on the joists. At the “−100” level, the thermal transmittance value is lower for this solution by 0.0031–0.0036 W/(m^2^·K) (1.36–1.62%) than for a junction with a self-supporting floor. This is due to the fact that the hemp–lime composites used as floor insulation differ in their thermal conductivity, so in the case of “a”, the wall insulation is in contact with a material with better thermal parameters (despite the fact that the thermal transmittance coefficient for floors in both variations is equal). However, with an increase of the level of the floor, the differences decrease, because the contact area between the floor and wall insulation increases. At the floor level “+100”, the situation is reversed. The thermal transmittance factor of the junction with the floor on joists is higher by 0.0003–0.0011 W/(m^2^·K) (0.66%–2.33%). It may be the impact of the timber joists that become a thermal bridge, along with enlarging their contact space with wall insulation. Statistical tests show the lack of significance of the differences in thermal transmittance values between both floor constructions (for *λ* = 0.080 W/(m·K) and other variables).

**Figure 8:**
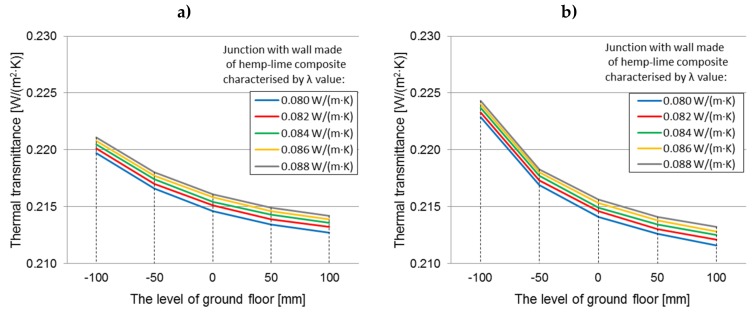
Averaged thermal transmittance coefficient of ground junction with “400c” wall filled with a hemp–lime composite with values of *λ* = 0.08–0.088 W/(m·K), depending on the floor level on the ground: (**a**) floor on joists, (**b**) self-supporting floor.

The graphs (Figure 8a,b) present the changes in the value of the average thermal transmittance coefficient for ground junctions with the wall thickness of 400 mm with the timber frame placed centrally with respect to the wall thickness filled with a hemp–lime composite with a thermal conductivity coefficient in the range 0.08–0.088 W/(m·K), depending on the floor level on the ground.

- The influence of the thermal conductivity values on the thermal transmittance values within each floor level (constant wall thickness and timber frame location)

The differences between the thermal transmittance values when using different hemp–lime composites (extreme thermal conductivity) on the example of a “400c” wall are 0.0014–0.0015 W/(m^2^·K) (0.64%–0.71%) for a junction with a floor on joists and also 0.0014–0.0016 W/(m^2^·K) (0.63%–0.76%) for a self-supporting floor, compared separately within each floor level. These differences are comparable regardless of the level of the floor on the ground, but they increase as the level of the floor decreases. As indicated by statistical analyses, with a constant wall thickness and location of the timber frame (in this case “400c”), the thermal conductivity has a positive effect on the thermal transmittance value. For the “400c” wall, the differences between the thermal transmittance values for extreme thermal conductivity are statistically significant for the junction with a floor on joists and for the junction with a self-supporting floor.

- The influence of the thermal conductivity values on the thermal transmittance values at extreme levels of floors

In the case of a self-supporting floor, the differences between the average thermal transmittance values for the ground junction are more pronounced (regardless of the thermal conductivity value of the wall composite). This is due to the inferior thermal performance of the thermal insulation in the self-supporting floor, despite the same thermal resistance of the floors in both variants and the analogous contact surfaces of the floor insulation with the wall insulation. The differences between the thermal transmittance values between the floor level “−100” and “+100” are 0.0069–0.0070 W/(m^2^·K) for the junction with the floors on joists and 0.0111–0.0130 W/(m^2^·K) for the junction with self-supporting floors and increase along with the thermal conductivity coefficient of the hemp–lime composite in the wall. The thermal transmittance value differences for junctions with the floor level “+100” and “−100” are statistically significant in both cases (floor on joists, self-supporting floor). A comparison of the extreme floor position (−100, 100) gives a difference in thermal transmittance values, which increases along with the lambda value.

**Figure 9:**
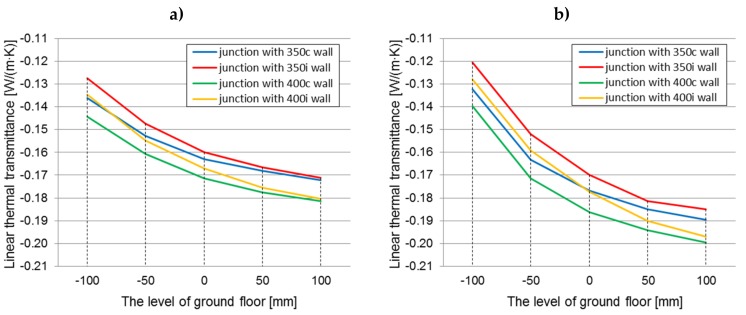
Linear thermal transmittance coefficient (calculated using the external dimensions) of the ground junction in all construction variants of walls filled with hemp–lime composite (*λ* = 0.080 W/(m·K)) dependent on the floor level on the ground: (**a**) floor on joists; (**b**) self-supporting floor.

The graphs (Figure 9a,b) show the changes in the linear thermal transmittance coefficient for ground junctions with all construction variants of the analyzed walls filled with hemp–lime composites with a thermal conductivity coefficient of 0.080 (W/m·K), dependent on the floor level.

- The influence of the timber frame location and the wall thickness on the linear thermal transmittance value within every floor level (constant thermal conductivity, 0.080 W/(m·K))

In both cases of the floor construction on the ground, better thermal parameters are exhibited by a junction with a timber frame placed centrally with respect to the wall thickness. The element with higher thermal conductivity (wood) is then surrounded by insulating material (hemp–lime composite). In the case of the floor on joists, linear thermal transmittance values are lower by 0.0011 W/(m·K)–0.0096 W/(m·K), while for the self-supporting floor, they are lower by 0.0027 W/(m·K)–0.0122 W/(m·K), relative to the junctions with the timber frame placed on the inside of the wall (taking into account both the wall thickness of 350 and 400 mm, but with the same value of λ = 0.080 W/(m·K)). The differences between linear thermal transmittance values in both cases decrease as the level of the floor increases.

In turn, comparing the thickness of the walls, lower linear thermal transmittance coefficients were obtained for walls, with the thickness of the composite layer in the wall equal to 400 mm (except for the level below “0” in the self-supporting floor, where the linear thermal transmittance factor for the junction with the wall “400i” was higher than for the junction with the wall “350c”). This relationship was observed in both cases of the floor construction on the ground. The differences in comparison to the junction with the ”350” wall were about 0.0072 W/(m·K)–0.0095 W/(m·K) (4.51%–5.94%) (floor A) and about 0.0072 W/(m·K)–0.0120 W/(m·K) (4.29%–6.28%) (floor B). The differences between linear thermal transmittance values in both cases decrease as the level of the floor increases.

The influence of the timber frame location is statistically significant and indicates lower linear thermal transmittance values for junctions with the timber frame placed centrally with respect to the wall thickness.

- The influence of the floor level on the linear thermal transmittance value in every wall thickness and location of the timber frame ((constant thermal conductivity, 0.080 W/(m·K))

The linear thermal transmittance coefficient decreases with the increase of the floor level, which means that the thermal bridge in the ground junction is reduced. The differences between the linear thermal transmittance value at “−100” and “+100” levels range from 0.0360 to 0.0456 W/(m·K) for a junction with a floor based on joists and from 0.0572 to 0.0689 W/(m·K) for a junction with a self-supporting floor, taking into account the wall thickness and the location of the timber frame. In both cases the greatest difference is for the “350i” wall and is the smallest for the “400c” wall. Along with raising the floor level, the envelope of the building is better insulated by enlarging the contact area of the floor and wall insulation, thereby limiting the heat escape path. In the case of the “−100” level, the contact of both insulations is the smallest, the heat freely penetrates through this connection, and in the case of the self-supporting floor, the heat flow will be increased due to the higher thermal conductivity of the hemp–lime composites in relation to the composite in the floor on the joists. The influence of the level of the floor on the linear thermal transmittance value of the junction is statistically significant for both types of floor construction.

- The influence of the type of floor construction (on joists or self-supporting) on the linear thermal transmittance value (constant: λ = 0.080 W/(m·K), variable: wall thickness, location of the timber frame, floor level)

A better thermal floor construction for the level below “0” is the floor on the joists. At the “−100” level, the linear thermal transmittance value is lower for this solution by 0.0037–0.0068 W/(m·K) (2.82%–5.66%) than for a junction with a self-supporting floor. However, while raising the level of the floor, the differences decrease, because the contact area between the floor and the wall insulation increases. At the floor level “+100”, the situation is reversed. The linear thermal transmittance-factor of the junction with the floor on the joists is higher by 0.0138–0.0182 W/(m·K) (8.05%–10.09%).

**Figure 10:**
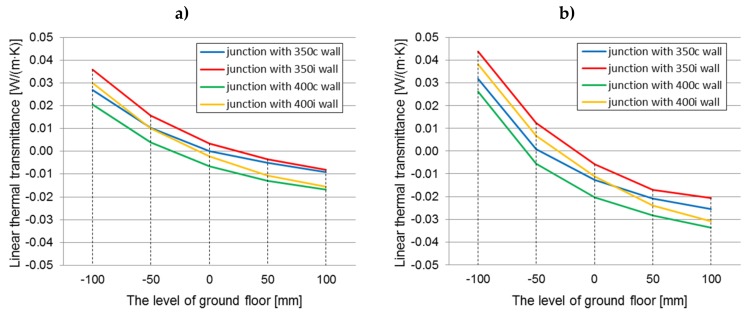
The linear thermal transmittance coefficient (calculated using the internal dimensions) of the ground junction in all construction variants of walls filled with hemp–lime composite (*λ* = 0.080 W/(m·K)), dependent on the floor level on the ground: (**a**) floor on joists; (**b**) self-supporting floor.

In order to compare the results, the changes in the linear thermal transmittance (calculated using the internal dimensions) coefficient dependent on the floor level, are presented in Figure 10a,b.

Higher values for linear thermal transmittance were obtained by calculating them according to Formula (2), using the internal dimensions. The values are 90.80%–128.06% higher for the floor on the joists and 83.14%–136.21% for the self-supporting floor. As a result of the calculations based on the external dimensions, the values at all floor levels are negative, while for the calculations based on the internal dimensions, the values at “+100”, and partially at “+50” and “0”, are positive. This is caused by the smaller dimensions of the partitions included in Formula (2).

**Figure 11:**
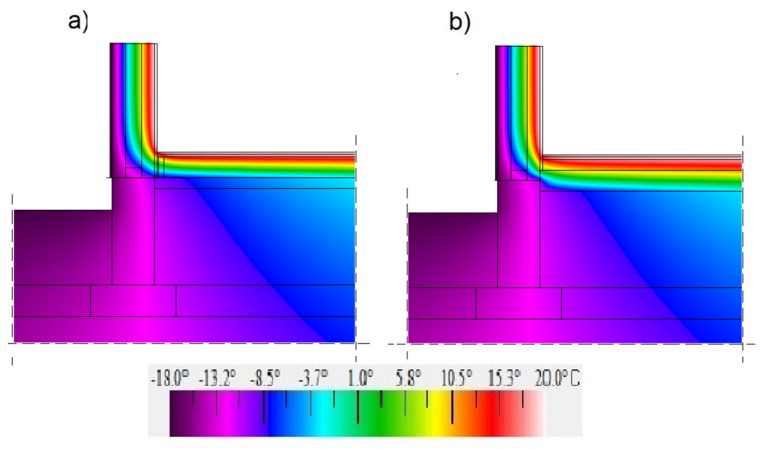
Temperature distribution in the ground junction with the “400c” wall filled with a hemp–lime composite (*λ* = 0.080 W/(m·K)) and with the floor on the ground at the adopted level “0”: (**a**) floor on joists; (**b**) self-supporting floor.

**Figure 12:**
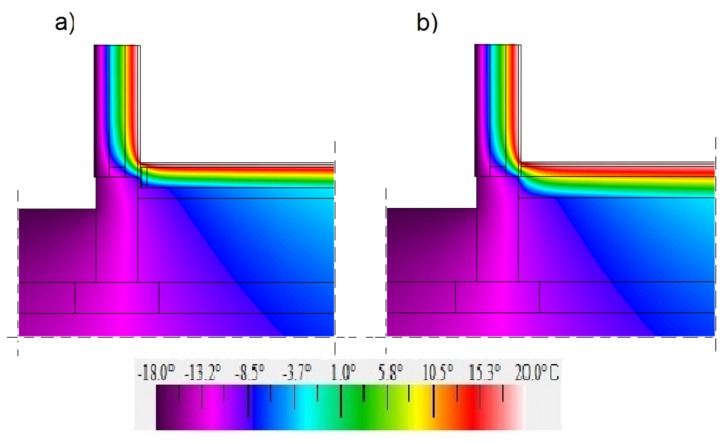
Temperature distribution in the ground junction with the “400c” wall filled with a hemp–lime composite (*λ* = 0.080 W/(m·K)) and with the floor on the ground at the adopted level “−100”: (**a**) floor on joists; (**b**) self-supporting floor.

**Figure 13:**
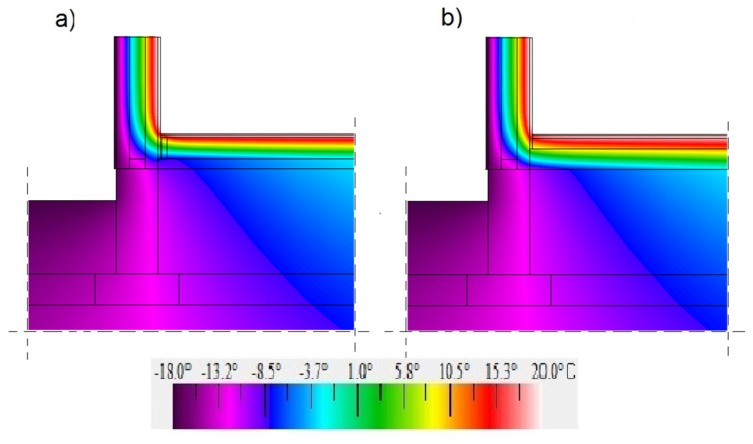
Temperature distribution in the ground junction with the “400c” wall filled with a hemp–lime composite (*λ* = 0.080 W/(m·K)) and with the floor on the ground at the adopted level “+100”: (**a**) floor on joists; (**b**) self-supporting floor.

Figure 11, Figure 12 and Figure 13 show maps in a colour scale illustrating the temperature distribution in the ground-level junction for the floor level on grounds “0”, “−100”, and “+100”.

Due to the higher thermal conductivity of insulation materials in the self-supporting floor (the hemp–lime composite and expanded clay aggregates) compared to the hemp–lime mixture in the floor variant on timber joists, a larger area of the insulation material in the self-supporting floor variant is subject to negative temperatures. The level of the floor on the ground determines the temperature on the entire contact surface between the floor and the wall insulation. In the case of a floor with a level of “−100”, the area of positive temperatures is the smallest. On the other hand, in the case of a floor with a “+100” level, the situation is reversed—the area of positive temperatures is the largest (among the analysed variants), which may improve the thermal comfort in the room.

### 3.2. Possibility of Water Vapour Condensation

The temperature factors for the described junctions are presented in Table 5. As the values did not differ significantly in the analysed range of the hemp–lime (wall mix) thermal conductivity, only the parameters for the hemp–lime mix with the thermal conductivity of 0.080 W/(m·K) are given.

The critical temperature factor f_0.25,max_ was calculated for the monthly mean relative humidity at the surface taken as 0.8, and it equals 0.78. It means that in some of the junctions (marked grey in Table 7), a risk of surface mould growth may occur. Furthermore, due to its construction type, the differences in the surface temperatures and temperature factors can be observed. In both cases of floor construction, the temperature factor increases along with the floor level due to the increasing contact surface of the floor insulation with the wall insulation. Higher temperature factors were observed in the case of a wall-self-supporting floor junction and in the case of centrally placed timber frame construction, which means that they are more beneficial in terms of an increased mould risk. The timber frame placed at the inner side of the wall causes a temperature drop on its surface, because the timber elements are characterized by a higher thermal conductivity than the hemp–lime composite. Lower temperature factors in the junctions with the floor on the timber joists may result from the fact that the wall insulation does not have direct contact with the floor insulation, only with the timber joist. This can also be seen as higher factors for the wall thickness of 400 mm than for 350 mm, but the differences are not significant, reaching about 0.01 °C.

## 4. Conclusions

This paper presents an analysis of the occurrence of thermal bridges in various types of the ground floor of a building with a timber frame structure filled with a hemp–lime composite. Both in the case of the floor on the ground of the joist structure and the self-supporting floor, the continuity of thermal insulation in the combination of the floor and the wall was preserved.

A thorough analysis of the obtained results enables one to formulate the following conclusions:

- All of the analyzed variables have a clear effect on the size of the thermal bridge and on the value of the average thermal transmittance coefficient in the ground junction
- Lowering the floor level by 100 mm in relation to the “0” level resulted in an increase in the thermal transmittance value by approx. 2.27%–2.83% for the floor on joists and about 3.79%–4.31% for the self-supporting floor, while the coefficient linear thermal transmittance was increased by 13.74%–20.30% and 22.16%–29.09%, respectively
- Raising the floor level by 100 mm in relation to the “0” level caused a decrease in the thermal transmittance value by about 0.76%–1.16% in the case of the floor on joists and about 1.07%–1.71% in the case of the self-supporting floor, while the linear thermal transmittance ratio was reduced by 5.04%–7.93% and 6.12%–11.06%, respectively
- The junction with a floor on timber joists has better thermal parameters than a junction with a self-supporting floor, in each of the analysed variants
- The differences in thermal conductivity are influenced by the quality of the contact zone of the wall and floor
- The differences in the values of the thermal transmittance of the junction between two variants of ground floors reach around 0.13%–1.67%, and the values of linear thermal transmittance factor were approximately 2.43%–10.13%; the greatest differences in results occur when the floor level is lowered by 100 mm
- Almost all the analysed junctions are free from surface condensation causing a risk of mould growth. This risk occurs in the case of a junction with a floor on joists located at “−100” and “−50”, and partly in the case of a self-supporting floor at the “-100” level (in junctions with walls in which the columns are placed on the inside). A preferable solution in this aspect is the floor on the ground and placing the timber frame centrally with relation to the wall thickness.
- Linear thermal transmittance was calculated using the steady state analyses, so its application in a dynamic simulation may be less accurate (however, it is accepted in the ISO 13790 standard).

## Figures and Tables

**Table 1:** Recipes of hemp–lime mixes (weight ratio), apparent density and thermal conductivity of the tested composites.

Composite Symbol	Binder: Hemp Shives Ratio	Binder: Water Ratio	Apparent Density [kg/m^3^]	Thermal Conductivity Coefficient [W/(m·K)]	Standard Deviation [W/(m·K)]
Wall mix A	1.4:1	1:1.45	362.5	0.080	± 0.002
Wall mix B	1.6:1	1:1.45	402.0	0.088	± 0.003
Floor mix A	1:1	1:1.5	238.0	0.065	± 0.002
Floor mix B	2.1:1	1:1.35	627.5	0.112	± 0.005

**Table 2:** The thermal conductivity of the main materials and elements.

Building Material/Element	Thermal Conductivity *λ* [W/(m·K)]
Hemp–lime mix (wall)	0.080–0.088
Hemp–lime mix (floor A)	0.112
Hemp–lime mix (floor B)	0.065
Timber construction element	0.16
OSB board	0.13
Lime plaster	0.70
Expanded clay aggregate	0.10
Concrete	1.30
Ballast of compacted sand	2.0
Ground soil	2.0

**Table 3:** Boundary conditions adopted in modelling.

Surface	Temperature [°C]	Surface Resistance [(m^2^·K)/W]	Description
Internal surface of the wall	+21	0.13	Heat flow horizontal, simplified *
Internal surface of the floor	+21	0.17	Heat flow downwards, simplified *
External (wall and ground)	−18	0.04	Simplified *
Internal (wall and floor)	+21	0.25	Condensation risk, simplified *
Cut-off planes	‒	‒	Adiabatic

* The simplified model means that convective and radiative heat exchange is described by one common surface resistance.

**Table 4:** Models for the averaged thermal transmittance and linear thermal transmittance: (1)—floor on timber joists, (2)—the self-supporting floor.

	Dependent Variable:			
	Averaged Thermal Transmittance		Linear Thermal Transmittance	
	(1)	(2)	(1)	(2)
λ	0.465 ***	0.221 ***	−2.688 ***	−2.570 ***
l	−0.0003 ***	−0.001 ***	−0.001 ***	−0.002 ***
$l^{2}$	0.00002 ***	0.00003 ***	0.0001 ***	0.0002 ***
w	−0.001 ***	−0.002 ***	−0.002 ***	−0.002 ***
f	−0.002 ***	0.002 ***	−0.014 ***	0.009 ***
λ·w	−0.007 ***	-	-	-
λ·f	0.041 ***	-	0.224 ***	-
l·w	-	-	−0.00002 ***	−0.00003 ***
l·f	−0.0001 ***	−0.0001 ***	−0.0004 ***	−0.0005 ***
$l^{2}\cdot f$	0.00000 ***	-	0.00002 ***	-
Constant	0.242 ***	0.263 ***	0.113 ***	0.090 ***
Observations	100	100	100	100
R^2^	0.999	0.997	0.998	0.995
Adjusted R^2^	0.999	0.997	0.998	0.995
Residual Std. Error	0.0002	0.0004	0.001	0.002
F Statistic	12,234.860 ***	4919.966 ***	5098.955 ***	2594.745 ***

Note: *** *p* < 0.01.

**Table 5:** Temperature factors f_0.25_ [°C].

No.	Type of Joint	Wall Thickness and the Location of the Timber Frame			
		350c	350i	400c	400i
1.	Timber joists floor level “+100”	0.86	0.86	0.87	0.86
2.	Timber joists floor level “+50”	0.80	0.80	0.81	0.81
3.	Timber joists floor level “0”	0.80	0.79	0.81	0.81
4.	Timber joists floor level “−50”	0.78	0.77	0.79	0.78
5.	Timber joists floor level “−100”	0.76	0.74	0.77	0.75
6.	Self-supporting floor Level “+100”	0.86	0.85	0.87	0.86
7.	Self-supporting floor Level “+50”	0.85	0.85	0.86	0.85
8.	Self-supporting floor Level “0”	0.84	0.83	0.85	0.84
9.	Self-supporting floor Level “−50”	0.83	0.81	0.83	0.82
10.	Self-supporting floor Level “−100”	0.79	0.77	0.79	0.78
